# Problems of subgroup analysis in randomized controlled trial

**DOI:** 10.1186/s12871-020-01105-8

**Published:** 2020-08-01

**Authors:** Hans-Joachim Priebe

**Affiliations:** Department of Anesthesiology and Critical Care, Medical Center University of Freiburg, Hugstetter Straße 55, 79095 Freiburg, Germany

**Keywords:** Trial registration, Outcome reporting, CONSORT guideline

## Abstract

Multiple subgroup analyses of the same data increase the risk of generating false positive findings. All outcomes and planned subgroup analyses should thus be prespecified and described in the original trial registry. When outcome changes during an ongoing trial seem justifiable, publications must disclose and explain such changes.

**To the Editor:**

The publication by Licker et al. [1] in BMC Anesthesiology raises several concerns. Foremost, all of the reported patients were included in a previous publication by the same group of authors [2]. The principal findings of both publications and of another very recent publication by the same group of authors [3] are basically identical, that is, glucose-insulin-potassium (GIK) infusion before start of cardiopulmonary bypass (CPB) improves LV function after open heart surgery. This creates the impression that three independent investigations [1–3] have documented a benefit of GIK-infusion during on-pump cardiac surgery. However, the publication by Licker et al. [1] reports findings in a post hoc defined subgroup of patients already included and analyzed in the first publication assessing the identical outcome, although the title states that this is a secondary analysis of the previously reported randomized controlled trial [2].

The authors claim that analysis of this subset of patients was pre-planned. This is an incorrect statement because it fundamentally contradicts the trial registration which reads: *“We will investigate the impact of short term GIK on the extent of myocardial injuries as well as on the left-ventricular systolic and diastolic function in 2 high-risk groups of cardiac surgical patients: Patients with cardiac dysfunction undergoing aortocoronary bypass surgery and patients with severe aortic stenosis.”* [4]. Furthermore, the authors define secondary study endpoints as” *other TEE parameters as well as hemodynamic parameters”*. However, the trial registration [4] lists as secondary outcome measures intraoperative systolic and diastolic cardiac function using transesophageal echocardiography (TEE), intraoperative hypo- and hyperglycemia and hypo- and hyperkalemia, 48 h postoperative serious cardiovascular adverse events (myocardial infarction, cardiac arrhythmia, low cardiac output, stroke), and intraoperative and 48 h postoperative serum troponin and creatine kinase concentrations. As a general principle, whenever changes from the original trial registration occur, they must be fully explained and reported to allow appropriate interpretation of results [5].

The authors neither mention nor critically discuss the considerable implications of a discrepancy between pre-specified and reported primary outcome measures. Accurate reporting of a pre-specified primary outcome in a subsequent publication is a critical component of clinical research, and incorrect reporting constitutes a major scientific flaw [6, 7]. Outcome misreporting increases the likelihood that reported differences are chance findings or are exaggerated [8]. Therefore, all outcomes and planned subgroup analyses should uniformly be prespecified and described in the original trial registry, and confidence intervals be provided for all outcomes to indicate the precision (uncertainty) of the estimate [9, 10]. The CONSORT (Consolidated Standards of Reporting Trials) statement even discourages, in general, multiple subgroup analyses of the same data because of the increased risk of generating false positive findings [7]. Unfortunately, reported primary outcomes in published randomized controlled trials frequently differ from those specified in the respective clinical trial registry [11, 12]. This includes publications in journals publicly endorsing the CONSORT guidelines on best practice in trial reporting which emphasize the importance of reporting all pre-specified outcomes [7, 12].

In the original publication [2], the a priori power calculation based on the primary outcome postcardiotomy ventricular dysfunction (PCVD) required 88 patients per group for adequate statistical power. Of those 88 patients, the present publication [1] reports selective findings in just 54 and 38 patients, respectively. In the absence of an a priori power calculation, it cannot be ruled out that the study is statistically underpowered. A statistical significance of interaction test was listed as one of 10 critical criteria for assessment of the credibility of a subgroup effect [9].

The CONSORT flow diagram was designed for the reporting of randomized trials [7]. Inclusion of such flow diagram in the publication [1] makes the reader believe that isolated coronary artery bypass graft (CABG) surgery, the combination of CABG surgery and aortic valve replacement (AVR), and poor quality or no TEE were exclusion criteria as per protocol. As this was not the case, presentation of a CONSORT flow diagram is inappropriate in this context. It is equally inappropriate and misleading to start the Discussion section with the words, *“In this randomized controlled trial ( …*)” when this publication does definitely not report findings of a randomized controlled trial.

Several of the statistically significant differences between groups in the absolute values of TEE variables reported in the publication [1] lie within the accuracy and precision of the applied methodology and are thus of questionable clinical relevance. Furthermore, presenting the same data as percent changes is unwarranted duplication of identical data and exaggerates the observed small changes in absolute values. Interpretation is exclusively based on *p*-values, a practice that is increasingly being discouraged [5, 6, 9]. Confidence intervals are not provided.

In conclusion, the present publication [1] is based on a previous study by the same authors [2]. It substantially overlaps in study hypothesis, study population, methods, data and conclusions, does not adhere to the pre-specified outcomes as defined in the trial registry, and does not introduce novel scientific aspects. Editors and reviewers can limit such shortcomings by, (a) ensuring that all results of an investigation are reported in a single comprehensive publication, (b) routinely consulting the trial registry when assessing a manuscript, and (c) demanding that authors report and analyze pre-specified outcomes [9, 10]. When outcome changes during an ongoing trial seem justifiable, publications must disclose and explain such changes [11].

## Data Availability

Not applicable.

## Abbreviations

LV: left ventricular
GIK: glucose-insulin-potassium
CPB: cardiopulmonary bypass
TEE: transesophageal echocardiography
CONSORT: Consolidated Standards of Reporting Trials
PCVD: postcardiotomy ventricular dysfunction
CABG: coronary artery bypass graft; aortic valve replacement
